# Regulatory γδ T cells induced by G-CSF participate in acute graft-versus-host disease regulation in G-CSF-mobilized allogeneic peripheral blood stem cell transplantation

**DOI:** 10.1186/s12967-018-1519-2

**Published:** 2018-05-25

**Authors:** Li Xuan, Xiuli Wu, Dan Qiu, Li Gao, Hui Liu, Zhiping Fan, Fen Huang, Zhenyi Jin, Jing Sun, Yangqiu Li, Qifa Liu

**Affiliations:** 1 Department of Hematology, Nanfang Hospital, Southern Medical University, Guangzhou, 510515 China; 2 Institute of Hematology, School of Medicine, Jinan University, Guangzhou, 510632 China

**Keywords:** Acute graft-versus-host disease, Allogeneic peripheral blood stem cell transplantation, Granulocyte colony-stimulating factor, Regulatory γδ T cells

## Abstract

**Background:**

The immunomodulatory effects of granulocyte colony-stimulating factor (G-CSF) on T cells result in a low incidence of acute graft-versus-host disease (aGVHD) in G-CSF-mobilized allogeneic peripheral blood stem cell transplantation (G-PBSCT). However, the exact mechanism remains unclear. Regulatory γδ T cells (γδTregs), characterized by the presence of TCRγδ and Foxp3, have aroused great concern in the maintenance of immune tolerance. We hypothesized that γδTregs might involve in the immunomodulatory effects of G-CSF mobilization.

**Methods:**

The expression and immunomodulatory function of γδTreg subsets in peripheral blood of donors before and after G-CSF treatment in vivo and in vitro were evaluated by flow cytometry and CFSE assays. To investigate the effects of γδTregs on aGVHD, the association between γδTreg subsets in grafts and aGVHD in recipients was estimated.

**Results:**

The proportions of Vδ1Tregs, CD27^+^Vδ1Tregs and CD25^+^Vδ1Tregs were significantly increased in peripheral blood after G-CSF treatment in vivo. γδTregs could be generated in vitro by stimulating with anti-TCRγδ in the presence of G-CSF. The immune phenotype, proliferation suppression function, and cytokine secretion of G-CSF-induced γδTregs were similar to that of transforming growth factor-β (TGF-β)-induced γδTregs. The clinical data demonstrated that the proportion of CD27^+^Vδ1Tregs in grafts was significantly lower in the patients who experienced aGVHD than in those who did not develop aGVHD (*P *= 0.028), and the proportions of other γδTreg subsets in grafts did not differ significantly between the two groups. The best cutoff value for CD27^+^Vδ1Treg proportion in grafts in prediction of aGVHD was 0.33%, with an area under the curve value of 0.725 (*P *= 0.043). Eight patients (26.7%) were classified as the low-CD27^+^Vδ1Treg group (< 0.33%), and 22 patients (73.3%) as the high-CD27^+^Vδ1Treg group (≥ 0.33%). The incidence of aGVHD was higher in the low-CD27^+^Vδ1Treg group than in the high-CD27^+^Vδ1Treg group (75.0% versus 22.7%, *P *= 0.028).

**Conclusions:**

G-CSF could induce the generation of γδTregs in vivo and in vitro, and γδTregs might participate in aGVHD regulation in G-PBSCT.

## Background

Nowadays granulocyte colony-stimulating factor (G-CSF) mobilized peripheral blood stem cell transplantation (PBSCT) has been more widely applied than bone marrow transplantation (BMT) due to its faster engraftment and practicability [1]. Although G-CSF-mobilized allogeneic PBSCT (G-PBSCT) contains more mature T cells, neither the incidence nor the severity of acute graft-versus-host disease (aGVHD) is higher compared with BMT [2, 3]. The protective effects of G-CSF against aGVHD might result from the immunoregulatory effects of G-CSF on T cells, including inhibiting T cell proliferation, polarizing T cells from the Th1 to Th2 phenotype, switching T cell cytokine secretion profile, and inducing CD4^+^CD25^+^Foxp3^+^T cells (regulatory T cells, Tregs) [4–7]. Recent studies have shown that Tregs with immunosuppressive function are not just confined to CD4^+^ T cells but also exist in CD8^+^ T and γδ T cell populations [8–11].

Regulatory γδ T cells (γδTregs), characterized by the presence of TCRγδ and a high level of Foxp3 expression, are a novel subset of γδ T cells with immunosuppressive effects [12–14]. γδTregs exist at very low frequencies in peripheral blood, and can be induced from peripheral blood mononuclear cells (PBMCs) in vitro in the presence of antigen stimulation and cytokines (transforming growth factor (TGF)-β1 and interleukin (IL)-2) [12, 14]. Recent studies have demonstrated that reduced numbers of γδTregs are correlated with the development of autoimmune diseases [12, 15, 16]. In addition, it has been confirmed that prophylactic infusion of γδTregs could reduce the incidence of GVHD in a mouse model [16]. Thus, γδTregs might be a new therapeutic target in autoimmune diseases. Our previous study has documented that G-CSF might change the distribution and clonality of the T cell receptors (TCRs) on γδ T cells, and this alteration might play a role in mediating GVHD in G-PBSCT [17]. Based on these results, we hypothesize that a possible mechanism of G-CSF inducing immune tolerance in G-PBSCT is that G-CSF induces γδTregs in grafts. To verify this hypothesis, we investigated the effects of G-CSF on γδTregs in vivo and in vitro, and explored the role of γδTregs in aGVHD in G-PBSCT recipients.

## Methods

### Samples

Peripheral blood (PB) was obtained from 30 healthy stem cell donors (13 female, 17 male; median age 33 years, range 12–56 years) before treatment and on the 5th day of treatment with G-CSF (Filgrastim, subcutaneous injection of 5 μg/kg/day; Kirin Brewery Co, Tokyo, Japan). G-CSF-primed PB (G-PB) grafts were harvested on the 5th day of treatment. The corresponding patients were 30 acute leukemia undergoing G-PBSCT from HLA-identical sibling donors. All donors and patients were willing to accept the trial after being informed, and all samples were obtained with consent. All of the procedures were conducted according to the guidelines of the Medical Ethics Committees of the Health Bureau of the Guangdong Province of China. This study was approved by the Ethics Committee of Nanfang Hospital and the Medical School of Jinan University.

### Flow cytometric analysis of γδTregs

γδ T cells in humans can be divided into two major groups, Vδ1 and Vδ2 T cells, depending on δ-chain usage [18, 19]. γδTregs have been reported to be characterized by the presence of TCR γδ and Foxp3 expression with a CD27^high^CD25^high^ phenotype [12, 14]. Therefore, γδTregs (Foxp3^+^γδ T cells) were classified as Vδ1Tregs (Foxp3^+^Vδ1 T cells) and Vδ2Tregs (Foxp3^+^Vδ2 T cells) in this study. FITC-conjugated anti-human TCR γδ (B1) and Vδ2 (B6), PE-conjugated anti-human CD25 (M-A251), PE-Cy7-conjugated anti-human CD3 (UCHT1), PerCP/Cy5.5-conjugated anti-human CD27 (M-T271), Alexa Fluor 647-conjugated anti-human Foxp3 (259D/C7) and the respective isotype control antibodies were purchased from BD Pharmingen. FITC-conjugated anti-human Vδ1 (TS 8.2) was obtained from Thermo Scientific. Purified no azide/low endotoxin mouse anti-human CD3 (UCHT1) and purified mouse anti-human TCR γδ (B1) were purchased from BD Pharmingen.

γδTreg subsets in PB before and after G-CSF in vivo treatment were analyzed by flow cytometry based on the above markers. PBMCs were first isolated from PB samples of healthy donors before and after G-CSF in vivo treatment by Ficoll-Hypaque density gradient centrifugation. Freshly isolated PBMCs were suspended in phosphate-buffered saline (PBS) containing 1% bull serum albumin. For the staining of surface antigens, cells were incubated with FITC-, PE-, PerCP/Cy5.5- and PE-Cy7-conjugated mAbs or their isotype controls for 30 min at 4 °C, respectively. For intracellular staining, cells were fixed and permeabilized with Perm/Fix solution, and finally stained with anti-Foxp3 Alexa Fluor 647 or isotype control according to the manufacturer’s instructions. Cells were assayed by BD FACS Canto™ II (BD Biosciences), and the acquired data were analyzed with FlowJo Software. Flow cytometric results were represented as percent positive.

### Induction of γδTregs in vitro

γδ T cells were first sorted by magnetic activated cell sorting (MACS, Miltenyi Biotec) from PBMCs of healthy stem cell donors, and then cultured in complete RPMI 1640 medium (supplemented with 2 mM l-glutamine, 10 mM HEPES, 10% fetal calf serum and 200 IU/ml recombinant human IL-2) in 6-well culture plates pre-coated with immobilized anti-human TCR γδ (1 μg/ml) for 12 days. In some groups, G-CSF (15 μg/ml) and/or TGF-β (2 ng/ml) were added at the beginning of the culture and replenished every 3 days. The culture system was grouped based on the differences in added cytokines, including (1) G-CSF, (2) TGF-β, (3) G-CSF + TGF-β and (4) blank control [(1)–(3) induction experimental groups]. After 3, 6, 9, and 12 days of culture, cells in the induction and control groups were harvested and analyzed by flow cytometry and real-time quantitative polymerase chain reaction (RQ-PCR). The percentages of γδTreg subsets were analyzed by flow cytometry. The expression levels of Foxp3, CD25, CTLA-4, GITR, STAT3, and Pias3 genes were examined by RQ-PCR.

### RQ-PCR

RNA was extracted from γδ T cells in the induction and control groups according to the manufacturer’s protocol (Trizol, Invitrogen, USA), and transcribed into the first single-stranded cDNA with random hexamer primers, using reverse transcriptase of the Superscript II Kit (Gibco, USA). The quality of cDNA was confirmed by reverse transcription PCR for β_2_ microglubin (β_2_M) gene amplification. The primer sequences of Foxp3, CD25, CTLA-4, GITR, STAT3, and Pias3 genes were listed in Table 1. RQ-PCR was performed in a volume of 20 ul containing 1 μl cDNA, 0.5 μM of each primer and 2.5 × RealMasterMix 9 μl (Tiangen, China). After the initial denaturation at 95 °C for 2 min, 45 cycles consisting of 95 °C 15 s, 60 °C 60 s and 82 °C 1 s for plate reading were performed using MJ Research DNA Engine Opticon 2 PCR cycler (BIO-RAD, USA). The relative mRNA expression levels of relative gene in each sample were calculated according to the comparative cycle time method [17, 20].

**Table 1:** PCR primers of target genes and β_2_M gene

Primer	Sequence
Foxp3-for	5′-CTGACCAAGGCTTCATCTGTG-3′
Foxp3-back	5′-ACTCTGGGAATGTGCTGTTTC-3
CD25-for	5′-ACCCTGAATTTGGCCTGCACTA-3′
CD25-back	5′-GACCTCCATCCCTTCTCCCTCT-3′
CTLA-4-for	5′-GCCCTGCACTCTCCTGTTTTT-3′
CTLA-4-back	5′-GGTTGCCGCACAGACTTCA-3′
GITR-for	5′-ACCCAGTTCGGGTTTCTCAC-3′
GITR-back	5′-CCAGATGTGCAGTCCAAGC-3′
STAT3-for	5′-CAGCAGCTTGACACACGGTA-3′
STAT3-back	5′-AAACACCAAAGTGGCATGTGA-3′
Pias3-for	5′-CTGGGCGAATTAAAGCACATGG-3′
Pias3-back	5′-AAAGCGTCGTCGGTAAAGCTC-3′
β_2_M-for	5′-TACACTGAATTCACCCCCAC-3′
β_2_M-back	5′-CATCCAATCCAAATGCGGCA-3′

### Cytokine measurements

The concentrations of interferon (IFN)-γ, IL-4, IL-10, IL-17, and TGF-β1 in the culture supernatant of three induction and control groups at days 3, 6, 9, and 12 were evaluated by Luminex analysis using Procarta Cytokine Assay Kits (Affymetrix) according to the manufacturer’s instructions [21, 22].

### Carboxyfluorescein diacetate (CFSE)-based suppression assay

CFSE assays to measure CD4^+^ T cell proliferation were performed as previously described [10, 12]. Autologous CD4^+^ T cells sorted by MACS (Miltenyi Biotec) were labeled with CFSE and used as responder cells. MACS-sorted γδ T cells before and after G-CSF treatment in vivo, or γδ T cells in the induction and control groups were used as effector cells. Responder cells (2 × 10^5^/well) were cultured alone or in combination with effector cells at a ratio of 1:1 in 12-well round-bottom plates pre-coated with anti-CD3 (1 μg/ml) in a final volume of 1 ml for 5 days. CFSE-unlabeled autologous CD4^+^ T cells were used as negative control, and CFSE-labeled CD4^+^ T cells alone were used as positive control. In the analysis of proliferation suppression effect of γδ T cells in the induction and control groups, to study the role that TGF-β played in the proliferation suppression of responder cells, CD4^+^ T cells were pretreated with TGF-β receptor inhibitor (10 μmol/l; LY364947, Cayman Chemical) for 1 h prior to the addition of effector cells in some experiments. After 5 days incubation, cells were harvested and analyzed by flow cytometry by gating on the CFSE-labeled cells, in which 10,000 events were collected. Data were analyzed with FlowJo Software. The percentages of divided responder T cells were compared.

### Cell counting kit-8 (CCK-8) assay

Cytotoxicity was measured with the CCK-8 assay kit (Dojindo, Japan). Sorted γδ T cells before and after G-CSF in vivo treatment, or γδ T cells in the induction and control groups were used as effector cells. Leukemia cell lines (Molt4, HL-60, and K562) or lymphoma cell lines (Raji and Daudi) were used as target cells. The effector cells were then mixed in a ratio of 1:1 with the target cells. A total of 10,000 cells were plated into each well of a 96-well plate, in which 10 μl CCK-8 was added to 100 μl of culture medium. The cells were subsequently incubated for 2–4 h at 37 °C, and the absorbance was measured at 450 nm. All the experiments were performed in triplicate.

### Statistical analysis

All data were analyzed using SPSS software, version 17.0. Results were represented as medians. Statistical significances for differences between the pre-G-CSF-treated and G-CSF-treated groups were assessed by the Wilcoxon matched pair test. Statistical significance among the induction and control groups was assessed by Kruskal–Wallis Test, and Bonferroni correction was used for pairwise comparisons. The cutoff value for γδTreg subsets in predicting aGVHD was determined using the receiver operating characteristics (ROC) curve analysis. Binary logistic regression analysis was used to estimate the association between G-PB graft cell subsets and aGVHD/relapse in recipients. aGVHD/relapse in recipients was considered as the dichotomous dependent variable, and the proportions of G-PB graft cell subsets were considered as the covariates. aGVHD was graded according to the literature [23]. *P *< 0.05 was considered statistically significant.

## Results

### G-CSF induces γδTregs in vivo

**Fig. 1:**
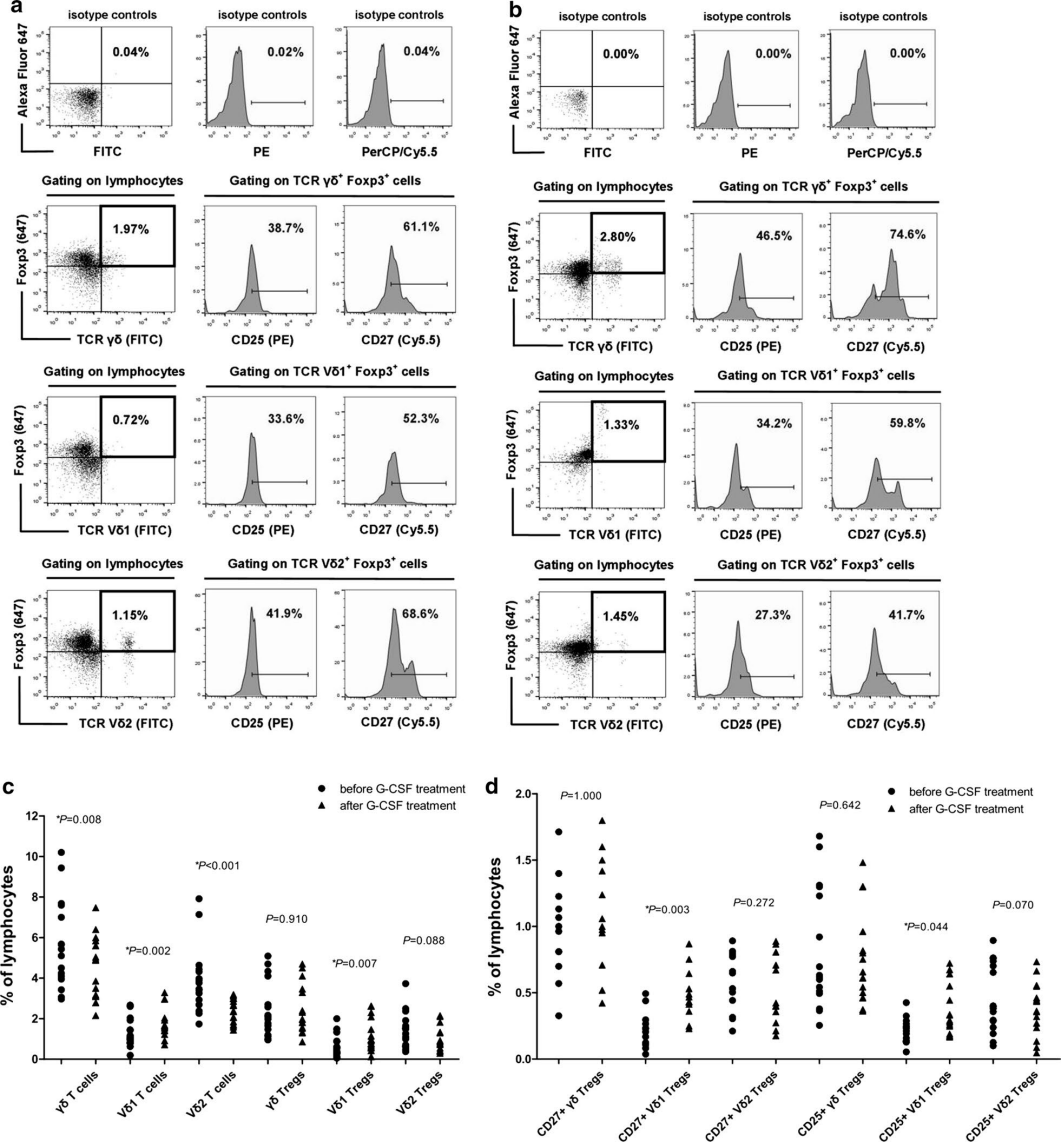
γδ T cell subsets in PBMCs of donors before and after G-CSF treatment in vivo. **a**, **b** CD27 and CD25 expression in γδTregs, Vδ1Tregs and Vδ2Tregs in PBMCs from 30 donors before (**a**) and after (**b**) G-CSF treatment in vivo (the figures are representative of one case). **c** Percentages of total γδ T cells, Vδ1 T cells, Vδ2 T cells, γδTregs, Vδ1Tregs and Vδ2Tregs in PBMCs of 30 donors before and after G-CSF treatment in vivo. **d** Percentages of CD27^+^γδTregs, CD27^+^Vδ1Tregs, CD27^+^Vδ2Tregs, CD25^+^γδTregs, CD25^+^Vδ1Tregs and CD25^+^Vδ2Tregs in PBMCs of 30 donors before and after G-CSF treatment in vivo. *P < 0.05

To investigate the effects of G-CSF on γδTregs, the proportions of total γδ, Vδ1, and Vδ2 T cells were first compared in PB from 30 donors before and after G-CSF treatment in vivo. The results demonstrated that there was an increase in Vδ1 T cell proportion (*P *= 0.002), and a decrease in the proportions of Vδ2 and total γδ T cells after in vivo administration of G-CSF (*P *< 0.001, *P *= 0.008), compared with that before G-CSF treatment (Fig. 1c). The Vδ1Treg proportion was significantly increased (*P *= 0.007), and the Vδ2Treg proportion displayed a decreased tendency (*P *= 0.088) after G-CSF treatment in vivo. The proportion of γδTregs did not significantly differ between the two groups (*P *= 0.910) (Fig. 1a–c). Additionally, we compared the CD27 and CD25 phenotypes in the γδTreg subsets, and observed a significant increase in the proportions of CD27^+^Vδ1Tregs and CD25^+^Vδ1Tregs after in vivo administration of G-CSF (*P *= 0.003, *P *= 0.044; Fig. 1a, b, d).

### G-CSF induces γδTregs in vitro

**Fig. 2:**
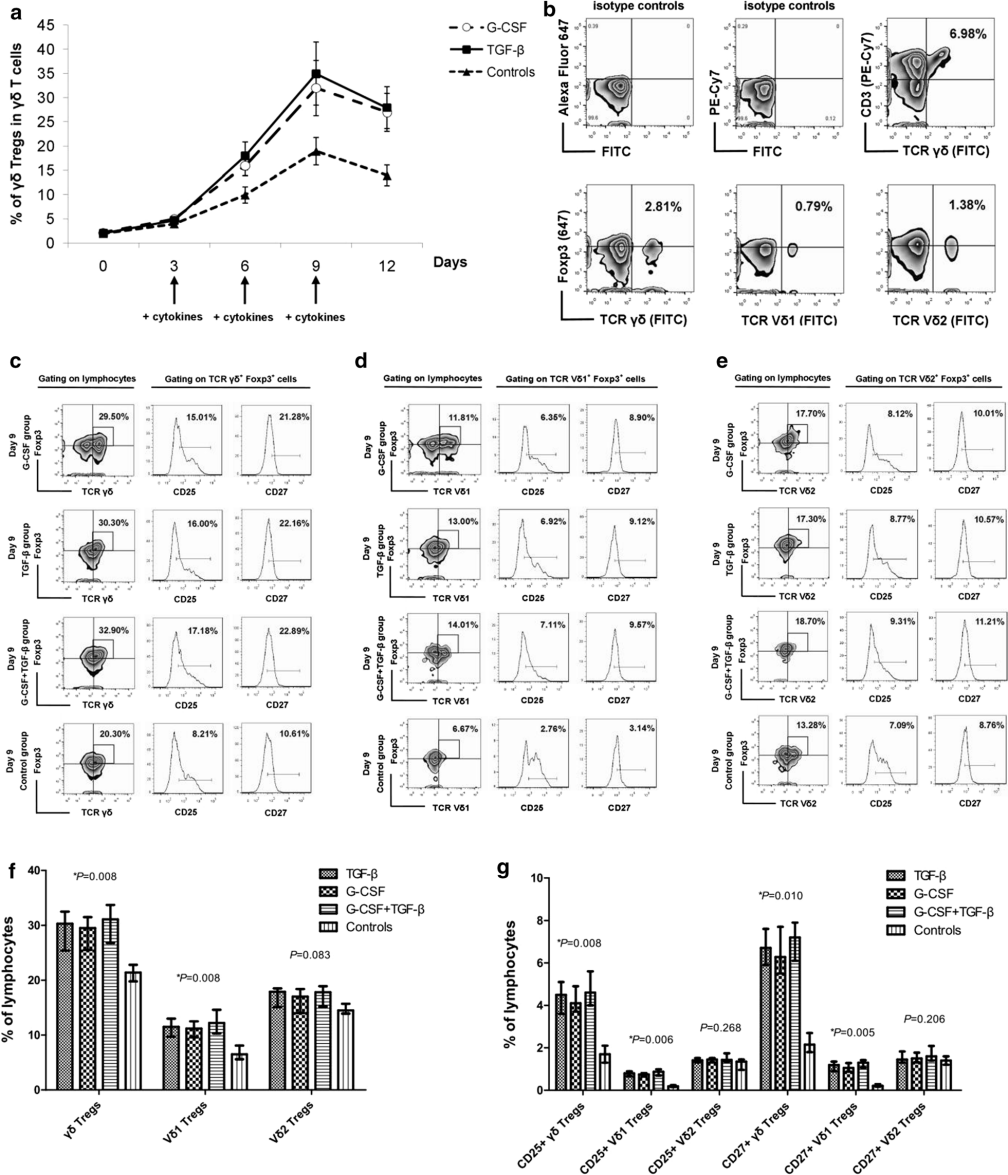
Proliferation of γδTreg subsets following anti-TCRγδ combined with G-CSF and/or TGF-β induction. **a** Kinetics of γδTreg proliferation following G-CSF or TGF-β induction during a 12-day culture period. **b** Proportions of γδTregs, Vδ1Tregs and Vδ2Tregs in freshly isolated PBMCs. **c**–**e** The expression of CD27 and CD25 phenotype on γδTregs (**c**), Vδ1Tregs (**d**) and Vδ2Tregs (**e**) in γδ T cells in the three induction and control groups on day 9 of culture. The data are representative of results from independent experiments of 5 healthy donors. **f** Proportions of γδTregs, Vδ1Tregs and Vδ2Tregs in γδ T cells in the three inductions and control groups on day 9 of culture. **g** Proportions of CD25^+^γδTregs, CD25^+^Vδ1Tregs, CD25^+^Vδ2Tregs, CD27^+^γδTregs, CD27^+^Vδ1Tregs and CD27^+^Vδ2Tregs in γδ T cells in the three inductions and control groups on day 9 of culture. *P < 0.05

To investigate whether G-CSF could induce γδTregs in vitro, MACS-sorted γδ T cells were cultured in the presence of anti-TCRγδ and G-CSF, and compared with TGF-β-induced γδTregs. As shown in Fig. 2a, b, sorted γδ T cells from the PBMCs of healthy donors rarely expressed Foxp3. After culture with anti-TCRγδ plus G-CSF or TGF-β, the γδTreg population was significantly increased. The expression of the γδTreg population reached its peak (approximately 30% or more) at the 9th day of culture and declined slightly afterwards. Therefore, the 9th day of culture was chosen as the observation point for evaluating the expression and function of γδTregs.

First, the proportions of the γδTreg subsets on day 9 were compared among the control and three induction groups. Compared with γδ T cells without induction, the proportions of γδTregs and Vδ1Tregs were significantly increased following G-CSF, TGF-β, or G-CSF + TGF-β induction (*P *= 0.008, *P *= 0.008) (Fig. 2f). The proportions of γδTregs and Vδ1Tregs in the G-CSF group were similar to that in the TGF-β and G-CSF + TGF-β group (*P *= 0.873, *P *= 0.336; *P *= 0.728, *P *= 0.309). The Vδ2Treg proportion did not significantly differ among the G-CSF, TGF-β, G-CSF + TGF-β and control groups (*P *= 0.083) (Fig. 2f). The CD25 and CD27 expression in the γδTreg subsets were then examined. Following G-CSF and/or TGF-β induction, the proportions of CD25^+^γδTregs and CD27^+^γδTregs were significantly increased (*P *= 0.008, *P *= 0.010) (Fig. 2c, g). These differences were mainly found in the Vδ1 T cells. The proportions of CD25^+^Vδ1Tregs and CD27^+^Vδ1Tregs were significantly increased after G-CSF and/or TGF-β induction (*P *= 0.006, *P *= 0.005) (Fig. 2d, g), whereas there was no significant difference in the proportions of CD25^+^Vδ2Tregs and CD27^+^Vδ2Tregs among the four groups (*P *= 0.268, *P *= 0.206) (Fig. 2e, g).

### G-CSF induces γδ T cells to express related molecules and cytokines

**Fig. 3:**
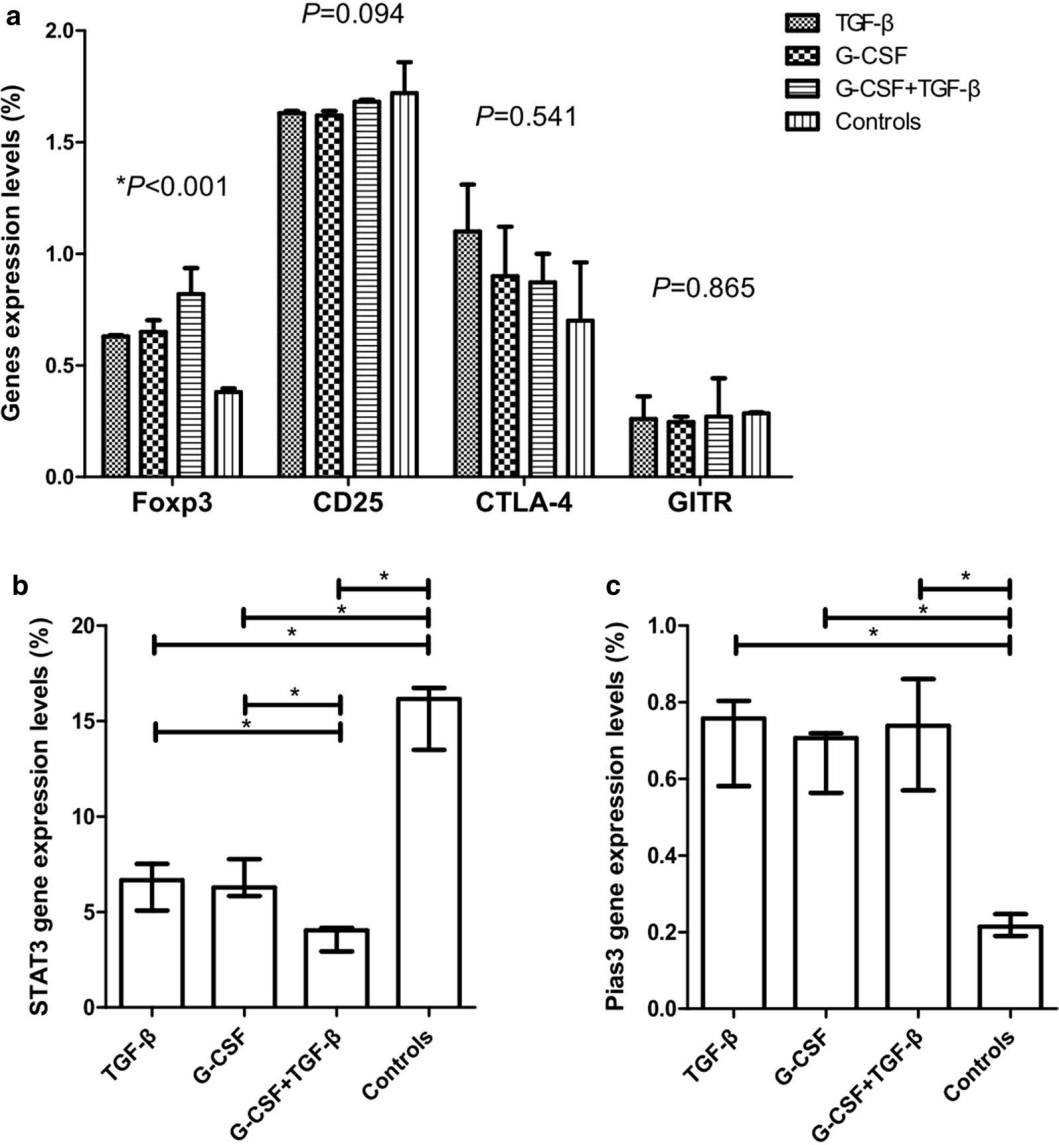
The mRNA expression of regulatory-related molecules in the inductions and control groups. The relative mRNA expression levels of regulatory-related molecules (**a**), STAT3 gene (**b**) and Pias3 gene (**c**) in the three inductions and control groups. *P < 0.05

γδTregs have been characterized to express a series of regulatory-related molecules, such as Foxp3, CD25, CTLA-4, and GITR [12, 13]. To compare differences in the expression of regulatory-related molecules in the induction and control groups, the expression of regulatory-related molecules at day 9 was analyzed by RQ-PCR. As illustrated in Fig. 3a, Foxp3 mRNA expression was greatly increased after G-CSF and/or TGF-β induction (*P *< 0.001), which was consistent with the higher proportion of γδTregs in the induction groups. The Foxp3 expression in the G-CSF group was similar to that in the TGF-β group (*P *= 0.951), but it was lower than that in the G-CSF + TGF-β group (*P *= 0.034). The γδ T cells in the induction and control groups had similar CD25, CTLA-4, and GITR expressions (*P *= 0.094, *P *= 0.541, and *P *= 0.865).

STAT3 is reported to promote the instability of natural Tregs and limit the generation of induced Tregs [24, 25]. Pias3 is the protein inhibitor of activated STAT3 [25]. Consequently, we speculated that STAT3-Pias-axis might affect the regulation of γδTregs, and compared the expression of STAT3 and Pias3 genes at day 9 in the induction and control groups. Pias3 expression was increased (*P *= 0.001), and STAT3 expression was decreased after G-CSF and/or TGF-β induction (*P *< 0.001) (Fig. 3b, c). Pias3 expression demonstrated no significant difference among the three induction groups (*P *= 0.806). The STAT3 expression in the G-CSF group was similar to that in the TGF-β group (*P *= 0.839), but higher than that in the G-CSF + TGF-β group (*P *= 0.019).

**Fig. 4:**
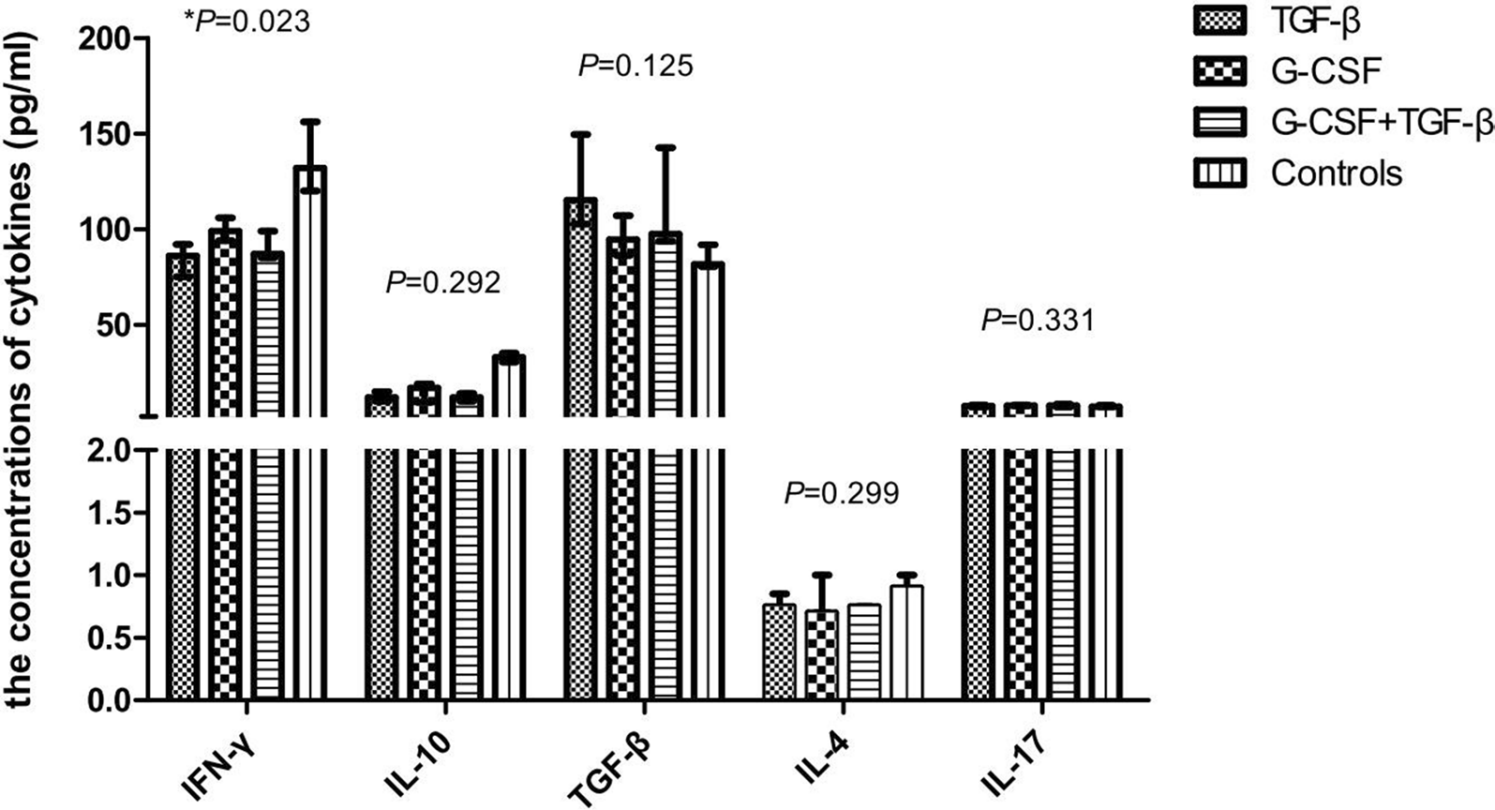
The concentrations of cytokines in the supernatant of the three induction and control groups. *P < 0.05

Moreover, the concentrations of IFN-γ, IL-4, IL-10, IL-17, and TGF-β1 in the culture supernatant at day 9 were compared among the control and induction groups. Supernatant cytokine measurements demonstrated that the concentration of IFN-γ was significantly decreased following G-CSF, TGF-β, or G-CSF + TGF-β induction (*P *= 0.023). γδ T cells in the control group produced higher amounts of IFN-γ than γδ T cells in the G-CSF, TGF-β, and G-CSF + TGF-β groups (*P *= 0.019, *P *= 0.006, and *P *= 0.014, respectively). A similar productions of TGF-β, IL-4, IL-10, and IL-17 were detected in γδ T cells in the three induction and control groups (*P *= 0.125, *P *= 0.299, *P *= 0.292, and *P *= 0.331, respectively) (Fig. 4).

### G-CSF-induced γδ T cells suppress CD4^+^ T cell proliferation and have certain cytotoxic effects on tumor cells

**Fig. 5:**
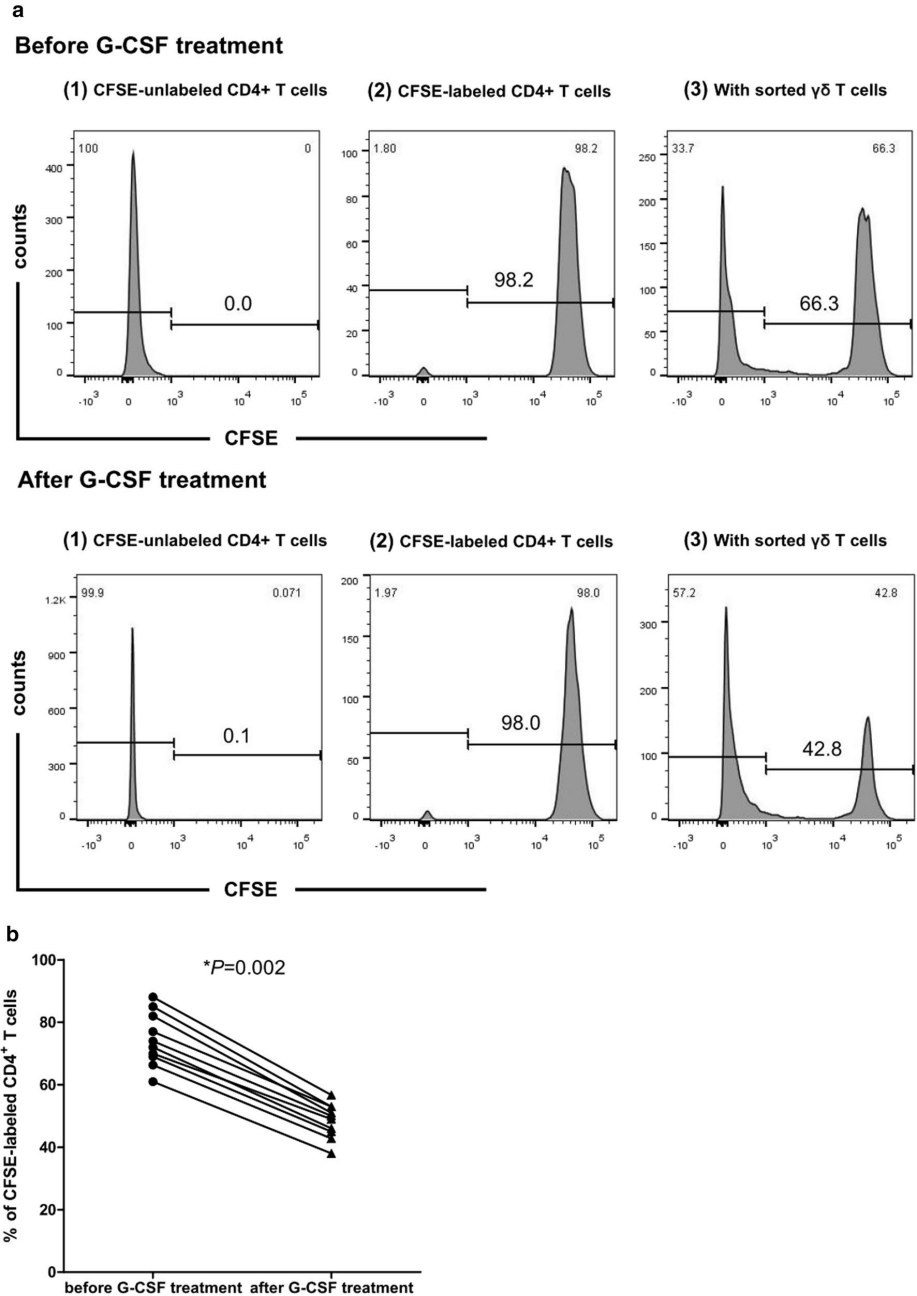
Sorted γδ T cells before and after G-CSF treatment in vivo exerted proliferation suppressive effects. **a** Proliferation of CFSE-labeled autologous peripheral blood CD4^+^ T cells (responders) co-cultured with sorted γδ T cells (effectors) before [(1)–(3)] and after [(4)–(6)] G-CSF treatment in vivo. (1), (4): negative controls (CFSE-unlabeled CD4^+^ T cells); (2), (5): positive controls (CFSE-labeled CD4^+^ T cells); (3), (6): experimental groups (CFSE-labeled CD4^+^ T cells + sorted γδ T cells). The data are representative of results from 10 healthy donors. (B) The scatter plot reflected the effect of sorted γδ T cells before and after G-CSF treatment in vivo on the proliferation rates of CFSE-labeled CD4^+^ T cells. *P < 0.05

**Fig. 6:**
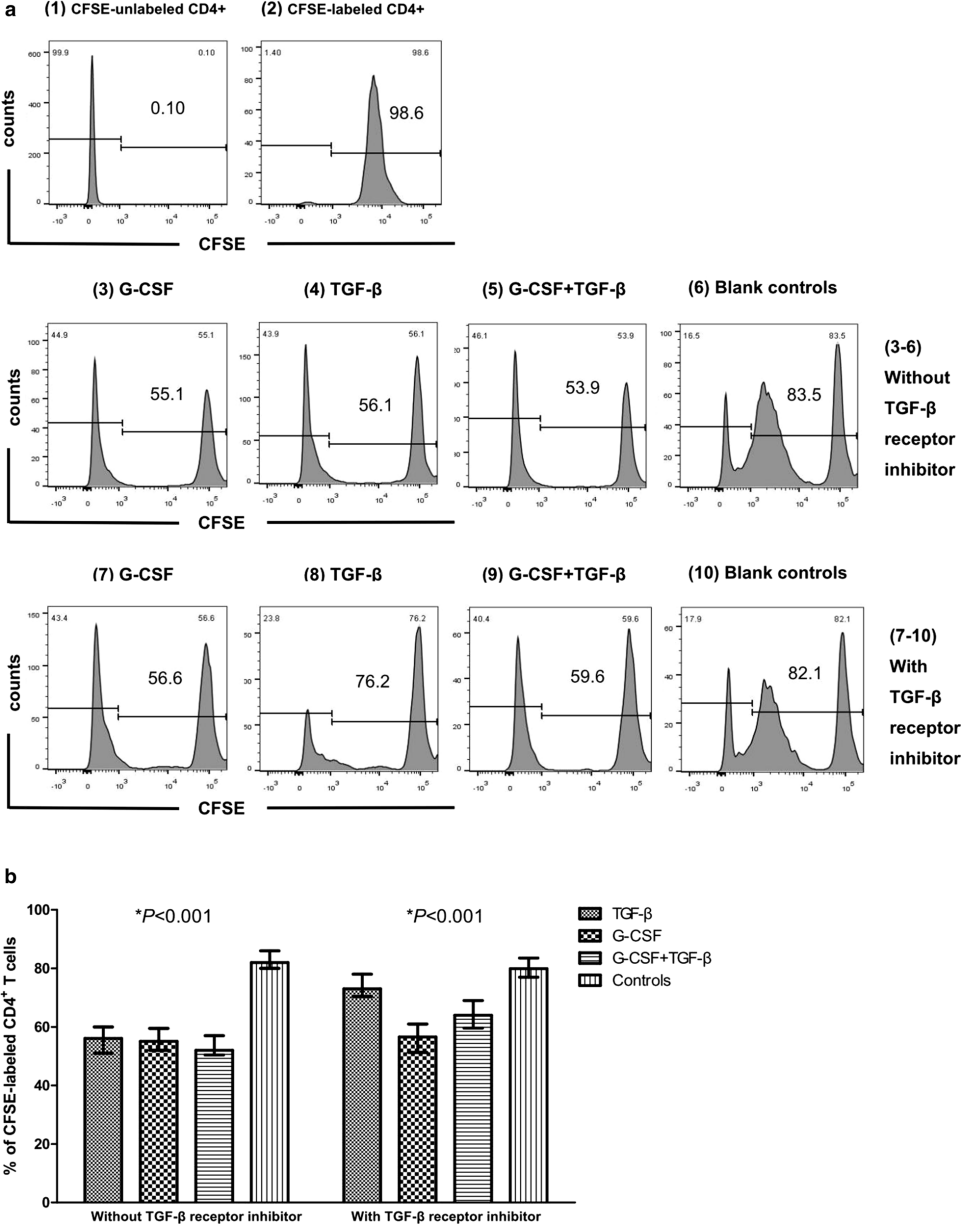
Anti-TCRγδ-stimulated γδ T cells exert suppressive effects on CD4^+^ T cell proliferation. **a** The proliferation of CFSE-labeled autologous peripheral blood CD4^+^ T cells (responders) co-cultured with anti-TCRγδ-stimulated γδ T cells on day 9 (effectors) without [(3)–(6)]/with [(7)–(10)] TGF-β receptor inhibitor. (1) negative control (CFSE-unlabeled responders); (2) positive control (CFSE-labeled responders); (3)–(10) experimental groups; (3), (7): CFSE-labeled responders +G-CSF-induced γδ T cells; (4), (8): CFSE-labeled responders + TGF-β-induced γδ T cells; (5), (9): CFSE-labeled responders + (G-CSF + TGF-β)-induced γδ T cells; (6), (10): CFSE-labeled responders + only anti-TCRγδ stimulated γδ T cells. The data are representative of results from independent experiments of 5 healthy donors. **b** The bar reflected the effect of anti-TCRγδ-stimulated γδ T cells in the three inductions and control groups at day 9 of culture on the proliferation rates of CFSE-labeled CD4^+^ T cells. *P < 0.05

**Fig. 7:**
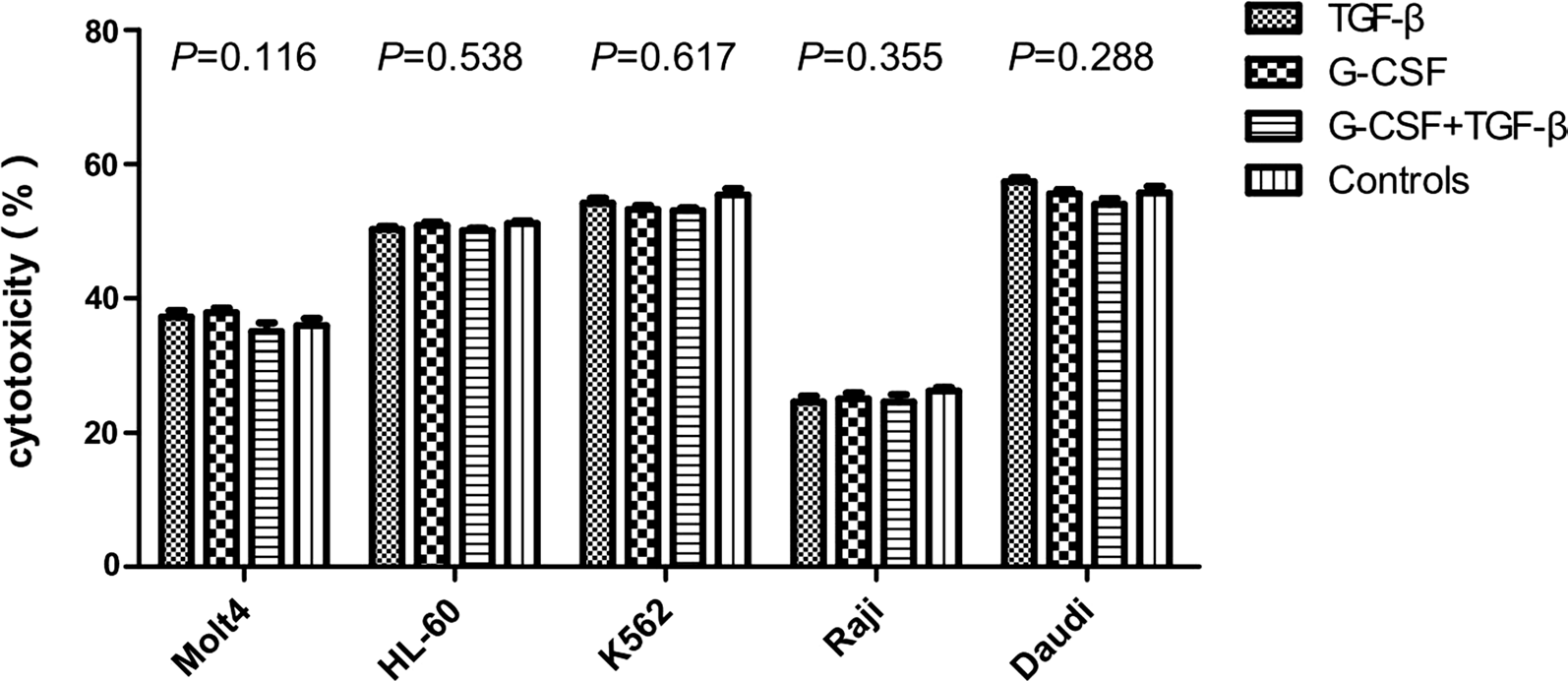
The cytotoxic effects of induced γδ T cells on leukemia and lymphoma cell lines. The cytotoxic effects of γδ T cells on leukemia cell lines (Molt4, HL-60, and K562) and lymphoma cell lines (Raji and Daudi) were not influenced by G-CSF, TGF-β, and G-CSF + TGF-β induction compared with the control group (*P *= 0.116, *P *= 0.538, *P *= 0.617, *P *= 0.355, and *P *= 0.288, respectively)

The proliferation suppressive and cytotoxic effects of sorted γδ T cells before and after G-CSF treatment in vivo were evaluated by CFSE and CCK-8 assays. The results demonstrated that sorted γδ T cells before and after treatment exerted certain suppression on the proliferation of CD4^+^ T cells, whereas sorted γδ T cells after G-CSF treatment had a higher suppressive effect on CD4^+^ T cells than that before treatment (*P *= 0.002, Fig. 5a, b). In addition, the proliferation suppressive and cytotoxic effects of γδ T cells in the induction and control groups were compared. As shown in Fig. 6a, b, G-CSF and/or TGF-β-induced γδ T cells had a similar suppressive effect on CD4^+^ T cells, which was a more significant suppressive effect than that of γδ T cells in the control group (*P *< 0.001). This result was in accordance with the above finding that γδ T cells in the induction groups expressed a higher proportion of γδTregs than that in the control group. We then used TGF-β receptor inhibitor to pre-treat CD4^+^ T cells and found that the suppressive effect could be reversed to some extent in the TGF-β and G-CSF + TGF-β group, whereas G-CSF combined with TGF-β receptor inhibitor could also have a certain suppressive effect. Moreover, the cytotoxic effects of γδ T cells on leukemia cell lines (Molt4, HL-60, and K562) and lymphoma cell lines (Raji and Daudi) were not influenced by G-CSF, TGF-β, and G-CSF + TGF-β induction compared with the control group (*P *= 0.116, *P *= 0.538, *P *= 0.617, *P *= 0.355, and *P *= 0.288, respectively) (Fig. 7).

### The proportion of CD27^+^Vδ1Tregs in grafts negatively correlates with aGVHD in G-PBSCT recipients

To explore the effects of γδTregs in grafts on aGVHD, 30 patients with acute leukemia in first complete remission (18 female, 12 male; median age 31 years, range 14–47 years) undergoing G-PBSCT from HLA-matched sibling donors were enrolled in this study. The primary diseases included acute myelocytic leukemia (AML, n = 18) and acute lymphoblastic leukemia (ALL, n = 12). The AML patients received busulfan (Bu) + cyclophosphamide (CY), and the ALL patients had total body irradiation (TBI) + CY conditioning regimen. Cyclosporine A plus methotrexate were administered in all of the 30 patients for GVHD prophylaxis. The 30 patients all achieved hematopoietic reconstitution. Of the 30 patients, 11 cases experienced aGVHD (6 with grade I and 5 with grade II) post-transplantation. Skin, gut and liver involvement was seen in 8, 2 and 1 patients, respectively. With a median follow up of 869 (range 147–981) days post-transplantation, 4 patients relapsed, 23 survived, and 7 died.

**Fig. 8:**
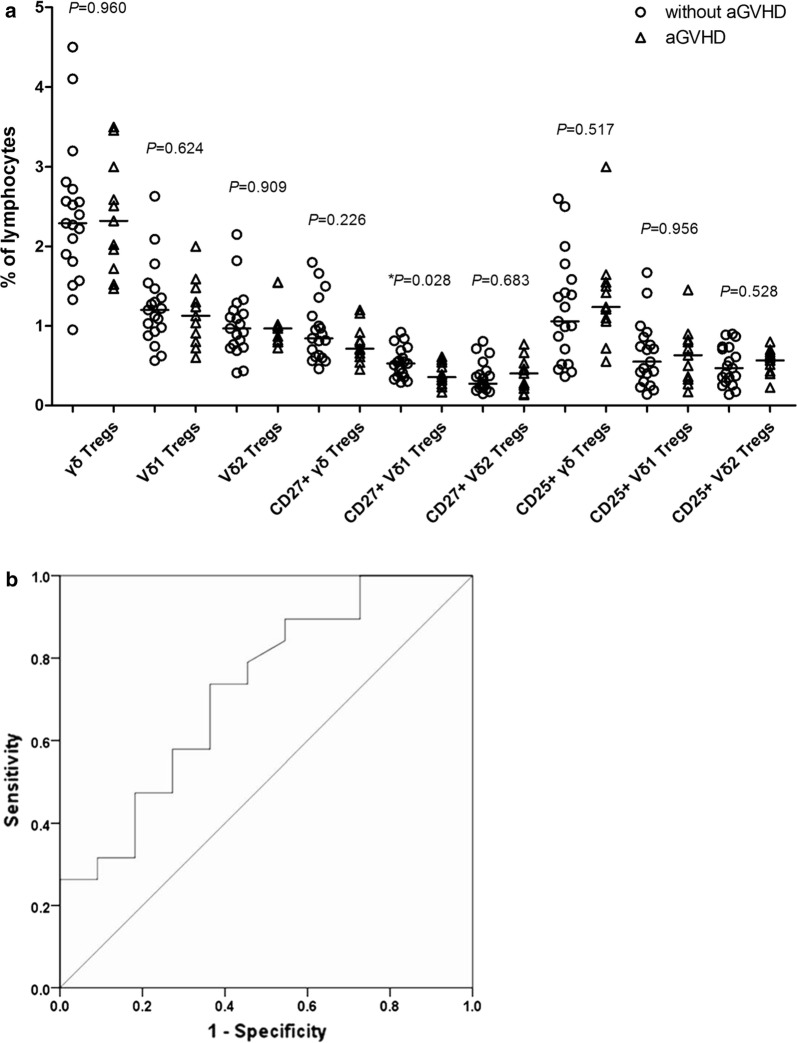
The correlation of γδTreg subsets in grafts and aGVHD in G-PBSCT recipients. **a** The proportions of γδTreg subsets in grafts in the patients who developed and did not develop aGVHD post-transplantation. **b** The ROC curve analysis for the optimal cutoff point of the proportion of CD27^+^Vδ1Tregs in grafts in prediction of aGVHD

We first analyzed the effects of γδTreg subsets in grafts (γδTregs, Vδ1Tregs, Vδ2Tregs, CD27^+^γδTregs, CD27^+^Vδ1Tregs, CD27^+^Vδ2Tregs, CD25^+^γδTregs, CD25^+^Vδ1Tregs and CD25^+^Vδ2Tregs) on the occurrence of aGVHD in G-PBSCT recipients. The results showed that the proportion of CD27^+^Vδ1Tregs in grafts was significantly lower in the patients who experienced aGVHD than in those who did not develop aGVHD (*P *= 0.028), and the proportions of other γδTreg subsets in grafts did not differ significantly between the two groups (all *P *> 0.05) (Fig. 8a). Then we performed the ROC curve analysis for the optimal cutoff point of CD27^+^Vδ1Treg proportion in grafts in prediction of aGVHD. The most discriminative cutoff value of CD27^+^Vδ1Treg proportion in grafts was 0.33% with an area under the curve value of 0.725 (*P *= 0.043, Fig. 8b). The sensitivity and specificity were 89.5 and 45.5%, respectively. According to the results of ROC analysis, we used the CD27^+^Vδ1Treg proportion in grafts 0.33% as the cutoff value. In terms of this cutoff value, 8 patients (26.7%) were classified as the low-CD27^+^Vδ1Treg group (< 0.33%), and 22 patients (73.3%) as the high-CD27^+^Vδ1Treg group (≥ 0.33%). The incidence of aGVHD was higher in the low-CD27^+^Vδ1Treg group than in the high-CD27^+^Vδ1Treg group (75.0% versus 22.7%, *P *= 0.028). In addition, there was no significant association between CD27^+^Vδ1Treg proportion and age, gender and disease type.

The association between other cell subsets in G-PB grafts and aGVHD in recipients was also analyzed. The median proportions of CD3^+^ T cells, CD3^+^CD4^+^ T cells, CD3^+^CD8^+^T cells, CD19^+^B cells, CD3^−^CD56^+^NK cells of lymphocytes in G-PB grafts were 70.45, 33.5, 27.1, 14.2 and 9.6%, respectively. G-PB graft cell subsets (CD3^+^T cells, CD3^+^CD4^+^ T cells, CD3^+^CD8^+^ T cells, CD19^+^B cells, CD3^−^CD56^+^NK cells and γδTreg subsets) were divided into two groups based on their medians. Simple effect analysis of binary logistic regression showed that the proportion of CD27^+^Vδ1Tregs in G-PB grafts was negatively associated with the incidence of aGVHD in recipients (*P *= 0.023, *r*_*s*_= − 0.563). Multivariate analysis also demonstrated that the higher proportion of CD27^+^Vδ1Tregs in G-PB grafts indicated a lower incidence of aGVHD in G-PBSCT recipients (*P *= 0.027, odds ratio = 0.033, 95% confidence interval 0.002–0.680). Nevertheless, the proportions of other cell subsets in grafts had no significant association with the incidence of aGVHD (all *P *> 0.05). In addition, we analyzed the association between γδTregs subsets in grafts and relapse in recipients. There was no association between the proportions of γδTreg subsets in grafts and leukemia relapse in recipients (all *P *> 0.05).

## Discussion

G-CSF-induced immune tolerance has been widely studied in G-PBSCT, but to our knowledge, this is the first report from the perspective of γδTregs. A few studies have suggested that γδTregs are implicated in the pathogenesis of GVHD [15, 16]. Our previous study has demonstrated that G-CSF might induce immune tolerance by influencing the clonality of TCRs on γδ T cells [17]. In this study, we investigated the effects of G-CSF on γδTregs and explored the role of γδTregs in aGVHD in G-PBSCT recipients. Our results suggested that G-CSF induced the generation of γδTregs in vivo and in vitro, and the higher proportion of CD27^+^Vδ1Tregs in grafts was associated with a lower incidence of aGVHD in G-PBSCT recipients. Therefore, we hypothesized that G-CSF inducing immune tolerance in G-PBSCT might be associated with the increase of γδTregs in grafts.

Under normal physiological conditions, γδTregs exist at very low frequencies in PB, and they are decreased in PB in some autoimmune diseases [12, 15]. Li et al. [12] reported that γδTregs could be induced from PBMCs in vitro by stimulating with anti-TCRγδ in the presence of TGF-β1. In this study, we found that γδTregs could not only be induced in vitro by stimulating with anti-TCRγδ in the presence of G-CSF but also in vivo by G-CSF alone. In addition, the immune phenotype, proliferation suppression function, and cytokine secretion of G-CSF-induced γδTregs were similar to that of TGF-β-induced γδTregs. The JAK/STAT3 signaling pathway might contribute to direct effects of G-CSF on T cells [24–26]. The interaction between G-CSF and its receptor promoted the phosphorylation of JAK proteins, which led to the phosphorylation and homodimerization of STAT3. The homodimerized STAT3 translocated to the nucleus and promoted the transcription of Pias3, resulting in enhancing Treg survival [24–26]. Our preliminary study found that STAT3 expression was decreased and Pias3 expression was increased after G-CSF treatment. Therefore, we speculated that G-CSF might induce γδTregs by increasing Pias3 expression and inhibiting STAT3 expression, which required further experimental verification. To explore whether G-CSF and TGF-β had synergistic effects in inducing γδTregs, sorted γδ T cells were cultured in vitro in the presence of G-CSF and TGF-β. Surprisingly, the combination of the two did not have a synergistic effect in inducing γδTregs. The reason of the result was not clear and needed further investigation. Interestingly, we observed that the proportion of γδTregs in the control group could reach up to 20%, which was consistent with Hua’s recent finding that Vδ1 T cells could be stimulated by anti-human TCR Vδ1 mAb in the absence of exogenous TGF-β1 [13]. We suggested that the results might be due to the effect of autocrine TGF-β1. The similar concentrations of TGF-β1 in the culture supernatant of three induction and control groups supported our suggestion. Additionally, as soluble cytokine TGF-β in culture might be involved in the proliferation suppression of the CD4 responder cells, we used TGF-β receptor inhibitor to pre-treat CD4^+^ T cells and found that the suppression effect could be reversed to some extent in TGF-β group, suggesting that cytokine TGF-β might also participate in the course. However, the suppression effect was not reversed in the G-CSF group, which indicated that G-CSF-induced γδTregs might have the proliferation suppressive effect on CD4^+^ T cells through the non-dependent TGF-β pathway.

γδ T cells consist of two major subsets in humans: Vδ1 and Vδ2. Vδ2 T cells predominate in PB, and Vδ1 T cells are frequently found in mucosal and intraepithelial tissue [27]. The two subsets are similar in many aspects, such as activation and expansion, but they have considerable differences in inherent gene expression. Vδ2 T cells tend to have a more inflammatory cell phenotype, and Vδ1 T cells have a more regulatory cell phenotype [28, 29]. In this study, we divided γδTregs into the Vδ1Tregs and Vδ2Tregs groups according to δ-chain usage, and analyzed the proportions of γδTreg subsets before and after G-CSF treatment in vivo. Our study demonstrated that the Vδ1Treg proportion was found to be increased in PB after G-CSF treatment in vivo. This result indicated that Vδ1 T cells had a more marked conversion to Foxp3 expression after G-CSF treatment.

γδ T cells have been shown to exert major histocompatibility complex -unrestricted natural cytotoxicity against different types of tumors such as neuroblastoma, esophageal carcinoma, and some subsets of lymphoma and leukemia [30–32]. Whether γδTregs have a cytotoxic effect on tumor cells remains unknown. Our study demonstrated that the proportion of γδTregs was significantly increased following G-CSF and/or TGF-β induction. The cytotoxic effects of γδ T cells on leukemia and lymphoma cell lines in the three induction and control groups did not significantly differ, indicating that γδTregs induced by G-CSF and/or TGF-β in vitro also had certain cytotoxic effects. Meanwhile, the clinical data demonstrated that the proportions of γδTregs in grafts had no significant association with leukemia relapse, suggesting that γδTregs in grafts had no effect on the function of graft-versus-leukemia.

Recent studies have demonstrated that γδTregs might play an important role in tumor immunity and autoimmunity [12, 33, 34], but their role in GVHD remains undefined. Hu et al. first reported that co-transplantation of γδTregs with human PBMCs in NOD/SCID mice could reduce the incidence of GVHD [16]. Subsequently, this group team demonstrated that the percentages of γδTregs were associated with the incidence and severity of chronic GVHD in patients undergoing allo-HSCT [15]. In this study, our results demonstrated that the proportion of CD27^+^Vδ1Tregs in grafts was negatively associated with the incidence of aGVHD in G-PBSCT recipients. It was reported that G-CSF could also expand other regulatory cell subsets like CD4^+^Foxp3^+^Tregs and myeloid-derived suppressor cells, which might contribute to the amelioration of aGVHD [35, 36]. Therefore, we deemed that G-CSF could affect not only Tregs but also γδTregs, and G-CSF inducing γδTregs in grafts might be an important way of G-CSF inducing immune tolerance. Although further prospective studies involving larger sample size are needed to validate the exact γδTreg graft dose that protects from aGVHD, our results strongly suggest γδTregs might be used as an evaluating indicator and therapeutic target for aGVHD in the future.

## Conclusions

To our best knowledge, we firstly demonstrated that G-CSF could induce the generation of γδTregs in vivo and in vitro. And lower incidence of aGVHD in G-PBSCT recipients might be in partly due to higher proportion of CD27^+^Vδ1Tregs after G-CSF mobilization. This finding might provide a theoretical basis and feasibility for employing γδTregs as a potential target for the prevention and treatment of aGVHD.

## Abbreviations

G-CSF: granulocyte colony-stimulating factor
PBSCT: peripheral blood stem cell transplantation
BMT: bone marrow transplantation
G-PBSCT: G-CSF-mobilized allogeneic PBSCT
aGVHD: acute graft-versus-host disease
Tregs: regulatory T cells
γδTregs: regulatory γδ T cells
PBMCs: peripheral blood mononuclear cells
TGF-β1: transforming growth factor-β1
IL-2: interleukin-2
TCRs: T cell receptors
PB: peripheral blood
G-PB: G-CSF-primed PB
PBS: phosphate-buffered saline
MACS: magnetic activated cell sorting
RQ-PCR: real-time quantitative polymerase chain reaction
β_2_M: β_2_ microglubin
IFN-γ: interferon-γ
CFSE: carboxyfluorescein diacetate
CCK-8: cell counting kit-8
ROC: receiver operating characteristics
AML: acute myelocytic leukemia
ALL: acute lymphoblastic leukemia
Bu: busulfan
CY: cyclophosphamide
TBI: total body irradiation
